# The Relation Between Daytime Sleepiness and Awake and Sleep Bruxism Report in Patients With Obstructive Sleep Apnoea

**DOI:** 10.1111/joor.70022

**Published:** 2025-07-24

**Authors:** Matteo Pollis, Frank Lobbezoo, Luca Guarda‐Nardini, Daniele Manfredini, Rosario Marchese‐Ragona

**Affiliations:** ^1^ Department of Medical Biotechnology, School of Dentistry University of Siena Siena Italy; ^2^ Department of Orofacial Pain and Dysfunction, Academic Centre for Dentistry Amsterdam (ACTA) University of Amsterdam and Vrije Universiteit Amsterdam Amsterdam the Netherlands; ^3^ Department of Orofacial Pain and Jaw Function, Faculty of Odontology Malmö University Malmö Sweden; ^4^ Unit of Oral and Maxillofacial Surgery Ca'Foncello Hospital Treviso Italy; ^5^ Department of Neurosciences, Otolaryngology Section University of Padova Padova Italy

**Keywords:** awake bruxism, daytime sleepiness, obstructive sleep apnoea, self‐report bruxism, sleep bruxism

## Abstract

**Objective:**

To assess the relationship between daytime sleepiness and both self‐reported awake bruxism (AB) and sleep bruxism (SB) in patients with different severities of obstructive sleep apnoea (OSA).

**Material and Methods:**

One hundred and seventy‐four participants (female = 33.9%; mean age [±SD] = 55.1 ± 12.3 years) with polygraphy‐confirmed OSA underwent a self‐reported assessment for both awake and SB and daytime sleepiness, using the BRUX scale questionnaire and Epworth sleepiness scale, respectively. Three BRUX scale sum scores were computed for each patient: total sum score, AB sum score and SB sum score. To assess OSA severity, the apnoea‐hypopnoea index and minimal oxygen saturation (MinSat) were considered. Correlations between daytime sleepiness, BRUX scale scores and OSA severity were assessed using Pearson's test. In addition, a multiple linear regression analysis model was used to assess the predictive effect of both self‐reported awake and SB and OSA severity on daytime sleepiness.

**Results:**

A significant, but weak correlation emerged between daytime sleepiness and the BRUX scale total sum score (*r* = 0.155; *p* < 0.05), the awake BRUX scale sum score (*r* = 0.174; *p* < 0.05) and MinSat (*r* = 0.194; *p* < 0.01). No significant correlations were found between OSA severity and any other variables. The multiple linear regression analysis showed that only the awake BRUX scale sum score had a positive predictive association with daytime sleepiness (*B* = 0.485; [95% CI = 0.076; 0.936]; *p* = 0.027).

**Conclusions:**

Within the limitations of this study, in individuals with OSA, self‐reported AB showed a predictive effect for daytime sleepiness, while no significant correlation between daytime sleepiness and OSA severity emerged.

## Graphical abstract

The present study enlightens the relevant issue that sleep disturbances may influence the onset of awake bruxism. Indeed, self‐reported awake bruxism, showing a significant correlation with daytime sleepiness, could be a sign of underlying tiredness and sleepiness in individuals with OSA.

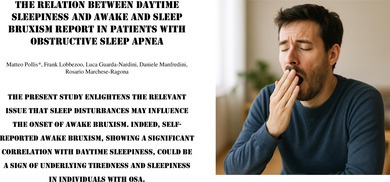

## 1 Introduction

Bruxism has received a lot of interest in the last decade thanks to a redefinition of the concept and its correlation with dental sleep medicine [1]. The International Consensus paper of 2018 provided two separate definitions of bruxism based on its circadian rhythm, viz. awake bruxism (AB) and sleep bruxism (SB) [2]. As reported by some systematic reviews, the prevalence of bruxism in the general population ranges from 8% to 31% for AB [3, 4], and from 9.7% to 15.9% for SB [3]. The main conceptual evolution concerned the fact that bruxism was no longer considered a disorder per se, but rather a potential sign of an underlying condition [2, 5]. However, this does not mean that the different forms of bruxism cannot have negative consequences in the stomatognathic system, the jaw‐muscle activity being potentially associated with mechanical tooth wear, masticatory muscle and articular pain, and prosthodontics complications [6, 7, 8, 9]. Within these premises, the existence of mutual correlations between various sleep‐related conditions (e.g., obstructive sleep apnoea [OSA]) suggested the need for early recognition of such conditions in the dental office, in order to intercept them and potentially prevent any negative consequences [10].

OSA is a breathing disorder featuring a total or partial repeated collapse of the upper airways during sleep [11]. These obstructive events can determine recurrent hypoxia and arousal from sleep, which is an abrupt shift in electroencephalographic frequency for at least 3 s, with at least 10 s of stable sleep prior to the event, that can lead to sleep fragmentation, daytime sleepiness and autonomic sympathetic system impairment [12]. Prevalence of OSA among the general population ranges from 9% to 38%, with even higher values in some risk groups, such as males, elderly people and obese individuals [13]. Furthermore, OSA has been shown to be a risk factor for chronic comorbidities, such as cardiovascular, neurological and metabolic diseases [14]. Thus, recent studies focus on deeper knowledge of the possible correlation with other conditions (e.g., AB and SB) for early diagnosis [10].

Concerning the correlation between OSA and SB, many observational studies found a higher prevalence of SB among individuals with OSA [15, 16, 17, 18, 19, 20] and a potential causal association with a protective effect of SB in individuals with OSA was even hypothesised in earlier investigations [21]. However, the lack of evidence of both severity‐based correlation and temporal correlation between SB and OSA events does not allow to confirm such causal relationship yet [22, 23]. On the other hand, it was asserted that sleep‐related arousal may justify the correlation between SB and OSA [15, 16, 24, 25, 26]. Indeed, although SB occurred as a physiological phenomenon in the cascade of events that characterised arousal from sleep [27], sleep disruption due to sleep apnoea may alter the sleep stage shifts and the frequency of transient arousals, in which SB events are more likely to occur [28].

In addition, the comprehension of the relationship, if at all existing, between AB and sleep disruption is even more limited and confused. It was speculated that AB is linked to psychological distress as a manifestation of anxiety traits and coping impairment [29, 30, 31, 32, 33]. Moreover, a correlation between sleep disturbances and psychological distress is largely reported [24]. Thus, the hypothesis of the existence of a correlation between sleep disturbances and AB may also be rational. Both AB and SB could be a sign of sleep disruption, and its detection may help to early diagnose underlying sleep‐related conditions and avoid the development of severe comorbidities.

Based on the above, the study hypothesis is that, since bruxism can be a sign of disruptive sleep, the report of bruxism could be related to daytime sleepiness, which is a well‐known symptom of sleep disturbances. The aim of this study is to assess the relationship between daytime sleepiness and both self‐reported awake and SB in patients with different severities of OSA.

## 2 Materials and Methods

### 2.1 Study Sample

This prospective study was carried out at the Sleep Apnea Center of University of Padova, Italy, from October 2018 to March 2020. At the first medical examination, each patient with suspected OSA underwent a cardiorespiratory polygraphy evaluation and completed two questionnaires for assessing both self‐reported bruxism and daytime sleepiness. Exclusion criteria were a history of treatment for OSA (e.g., upper airway or maxillofacial surgery, continuous positive airway pressure [CPAP]), ongoing treatment with mandibular advancement device (MAD), craniofacial abnormalities, head–neck neoplasia and age < 18. Data collection was forcedly interrupted by the Coronavirus disease (COVID‐19), and a sample of 174 patients (115 males, 59 females; mean age = 55.1 ± 12.3) was finally included in the study.

The study was approved by the Advisory Board of the School of Dentistry, University of Padova (#00457‐D87; 26/09/2018). All individuals gave their informed consent to take part in the study in accordance with the Helsinki Declaration.

### 2.2 Polygraphy Examination

Demographic variables such as age, sex and body mass index (BMI) were collected before the polygraphy examination.

Each individual underwent overnight single‐night home sleep testing with cardiorespiratory polygraphy (Embletta MPR, Natus Medical Incorporated, Middleton, WI, USA) which included monitoring of heart rate, nasal air‐flow, snoring, chest wall and abdominal excursion, oxygen saturation and body position. The recording time was set at 8 h, starting at the patient's bedtime. All signals were recorded and fed into a personal computer; the data were analysed using the Embletta software. According to the American Academy of Sleep Medicine (AASM) criteria [34], apnoea was defined as a > 90% drop in the flow amplitude with respect to the pre‐event baseline for at least 10 s, while a ≥ 30% drop in the flow amplitude with respect to the pre‐event baseline for at least 10 s, associated with a minimal reduction of 3% of oxygen saturation or an arousal event, was considered hypopnoea. apnoea–hypopnoea index (AHI) and minimal oxygen saturation (MinSat) were assessed.

### 2.3 Questionnaires

The BRUX scale questionnaire was used to assess self‐reported bruxism [35]. The BRUX scale consists of four questions that assess how often patients clench or grind their teeth during sleep or while awake (How often do you clench/grind your teeth during sleep/while awake?). Possible answers are based on a 5‐point Likert‐type ordinal scale (i.e., 1 = ‘never’; 2 = ‘occasionally’; 3 = ‘often’; 4 = ‘very often’; 5 = ‘always’). Three BRUX scale sum scores were computed for each patient: total sum score, AB sum score and SB sum score.

Daytime sleepiness was assessed with Epworth Sleepiness Scale (ESS) [36]. ESS investigates the risk of falling asleep in eight different situations, with response options ranging from ‘absence of risk’ (i.e., 0 point) to ‘mild’ (i.e., 1 point), ‘moderate’ (i.e., 2 points) and ‘high’ (i.e., 3 points) risk of sleepiness.

### 2.4 Statistical Analysis

After data collection, a descriptive analysis was performed for all items. The Shapiro test was performed to verify the normality of the data.

To assess the correlations between daytime sleepiness, self‐reported AB and SB, and OSA severity, Pearson's correlation test was used. The null hypothesis was that correlation does not exist, and a threshold of *p* < 0.05 was set to reject the null hypothesis. Both the BRUX scale total sum score and the awake and SB sum scores were included.

In addition, multiple linear regression analysis was used to assess the predictive effect of both self‐reported awake and SB and OSA severity for daytime sleepiness. The independent variables ‘Age’, ‘BMI’, ‘AHI’, ‘MinSat’, ‘BRUX scale total sum score’, ‘awake BRUX scale sum score’ and ‘sleep BRUX scale sum score’ were included in the backword regression model. All statistical procedures were performed with the software SPSS 29.0 (IBM Corp, Armonk, NY, USA).

## 3 Results

### 3.1 Descriptive Analysis

The sample included 174 participants (female = 59; 33.9%). The mean age (±SD) of the sample was 5.1 (±12.3) years with an age range of 19–78. Most of the sample was overweight with a mean BMI (±SD) of 27.8 (±4.6). Mean ESS (±SD) was 6.5 (±3.9).

Mean AHI (±SD) and mean MinSat (±SD) were 25.7 (±18.6) and 79.6 (±10.6), respectively. Males reported significantly higher AHI (28.3 ± 19.9) than females (21.1 ± 15.0) (*p* < 0.05), while no significant differences for MinSat emerged. The mean BRUX scale score of the sample was 2.05 (±2.8), with no significant differences between the two genders (males = 1.9 ± 2.7; female = 2.3 ± 3.0) (*p* > 0.05).

### 3.2 Correlation and Multiple Linear Regression Analysis

Correlation analysis between BRUX scale sum scores, ESS and respiratory indexes showed a significant, albeit weak, correlation between ESS and both BRUX scale total sum score (*r* = 0.155; *p* = 0.04), awake BRUX scale sum score (*r* = 0.174; *p* = 0.02) and MinSat (*r* = 0.194; *p* < 0.01). However, OSA severity did not show any correlation with any variables (*p* > 0.05) (Table 1). In the backward multiple linear regression analysis, it was found that only the awake BRUX scale sum score had a positive predictive association with daytime sleepiness (*B* = 0.485 [95% CI = 0.076; 0.936]; *p* = 0.027) (Table 2).

**TABLE 1:** Pearson's correlation between self‐report bruxism (BRUX scale total sum score, awake and sleep BRUX scale sum scores), daytime sleepiness (ESS) and respiratory indices (AHI and MinSat).

	ESS	Age	BMI	AHI	MinSat	Sex	BRUX scale total sum score	AB BRUX scale sum score	SB BRUX scale sum score
ESS									
Pearson's correlation	1	−0.047	−0.03	−0.089	0.074	0.143	0.155	0.175	0.107
Sig. (two‐tailed)	—	0.269	0.347	0.12	0.165	0.03*	0.02*	0.011*	0.08
*N*	174	174	174	174	174	174	174	174	174
Age									
Pearson's correlation	−0.047	1	0.073	0.092	−0.19	0.1	−0.136	−0.19	−0.067
Sig. (two‐tailed)	0.269	—	0.169	0.113	0.006**	0.095	0.037*	0.006**	0.188
*N*	174	174	174	174	174	174	174	174	174
BMI									
Pearson's correlation	−0.03	0.073	1	0.304	−0.449	−0.076	−0.122	−0.063	−0.136
Sig. (two‐tailed)	0.347	0.169	—	< 0.001**	< 0.001**	0.159	0.055	0.204	0.036*
*N*	174	174	174	174	174	174	174	174	174
AHI									
Pearson's correlation	−0.089	0.092	0.304	1	−0.54	−0.178	−0.022	−0.092	0.032
Sig. (two‐tailed)	0.12	0.113	< 0.001**	—	< 0.001**	0.009**	0.385	0.114	0.337
*N*	174	174	174	174	174	174	174	174	174
MinSat									
Pearson's correlation	0.074	−0.19	−0.449	−0.54	1	0.097	0.125	0.194	0.048
Sig. (two‐tailed)	0.165	0.006**	< 0.001**	< 0.001**	—	0.101	0.05	0.005*	0.266
*N*	174	174	174	174	174	174	174	174	174
Sex									
Pearson's correlation	0.143	0.1	−0.076	−0.178	0.097	1	0.077	0.056	0.076
Sig. (two‐tailed)	0.03*	0.095	0.159	0.009*	0.101	—	0.155	0.233	0.161
*N*	174	174	174	174	174	174	174	174	174
BRUX scale total sum score									
Pearson's correlation	0.155	−0.136	−0.122	−0.022	0.125	0.077	1	0.812	0.91
Sig. (two‐tailed)	0.02*	0.037*	0.055	0.385	0.05	0.155	—	0	0
*N*	174	174	174	174	174	174	174	174	174
AB BRUX scale sum score									
Pearson's correlation	0.175	−0.19	−0.063	−0.092	0.194	0.056	0.812	1	0.496
Sig. (two‐tailed)	0.011*	0.006**	0.204	0.114	0.005*	0.233	0	—	0
*N*	174	174	174	174	174	174	174	174	174
SB BRUX scale sum score									
Pearson correlation	0.107	−0.067	−0.136	0.032	0.048	0.076	0.91	0.496	1
Sig. (two‐tailed)	0.08	0.188	0.036*	0.337	0.266	0.161	0	0	—
*N*	174	174	174	174	174	174	174	174	174

** Correlation is significant at the 0.01 level (two‐tailed).

* Correlation is significant at the 0.05 level (two‐tailed).

**TABLE 2:** Multiple linear regression analysis (backward model) between daytime sleepiness (ESS) and demographic (i.e., age, BMI), OSA‐related (i.e., AHI, MinSat) and self‐report bruxism‐related (i.e., BRUX scale total sum score, awake BRUX scale sum score and sleep BRUX scale sum score) variables.

Model	Unstandardized coefficients		Standardised coefficients	*t*	Sig.	95% confidence interval for *B*	
	*B*	SE	Beta			Lower bound	Upper bound
1							
(Constant)	6039	4637		1302	0.195	−3116	15 194
Age	−0.008	0.025	−0.025	−0.324	0.746	−0.057	0.041
BMI	0.011	0.074	0.013	0.146	0.884	−0.135	0.156
AHI	−0.011	0.019	−0.053	−0.584	0.56	−0.049	0.027
MinSat	0.002	0.037	0.005	0.049	0.961	−0.07	0.074
Sex	1039	0.64	0.126	1622	0.107	−0.225	2303
SB BRUX scale sum score	0.056	0.183	0.027	0.306	0.76	−0.306	0.418
AB BRUX scale sum score	0.418	0.262	0.144	1595	0.113	−0.099	0.935
2							
(Constant)	6235	2276		2.74	0.007	1743	10 728
Age	−0.008	0.025	−0.026	−0.334	0.738	−0.057	0.041
BMI	0.009	0.068	0.011	0.138	0.891	−0.126	0.145
AHI	−0.012	0.017	−0.055	−0.681	0.497	−0.045	0.022
Sex	1039	0.638	0.126	1628	0.106	−0.221	2299
SB BRUX scale sum score	0.055	0.182	0.027	0.304	0.762	−0.305	0.416
AB BRUX scale sum score	0.42	0.258	0.145	1629	0.105	−0.089	0.929
3							
(Constant)	6476	1459		4438	< 0.001	3595	9356
Age	−0.008	0.025	−0.025	−0.329	0.742	−0.057	0.04
AHI	−0.011	0.016	−0.052	−0.673	0.502	−0.043	0.021
Sex	1037	0.636	0.126	1.63	0.105	−0.219	2294
SB BRUX scale sum score	0.052	0.18	0.025	0.287	0.775	−0.303	0.406
AB BRUX scale sum score	0.422	0.257	0.146	1643	0.102	−0.085	0.929
4							
(Constant)	6486	1455		4459	< 0.001	3615	9358
Age	−0.008	0.025	−0.025	−0.326	0.745	−0.056	0.04
AHI	−0.01	0.016	−0.05	−0.649	0.517	−0.042	0.021
Sex	1.05	0.633	0.127	1659	0.099	−0.2	2.3
AB BRUX scale sum score	0.458	0.222	0.158	2062	0.041*	0.019	0.897
5							
(Constant)	6056	0.61		9929	< 0.001	4852	7261
AHI	−0.011	0.016	−0.052	−0.686	0.494	−0.043	0.021
Sex	1024	0.626	0.124	1635	0.104	−0.212	2.26
AB BRUX scale sum score	0.472	0.218	0.163	2168	0.032	0.042	0.902
6							
(Constant)	5738	0.395		14 515	< 0.001	4958	6518
Sex	1098	0.616	0.133	1784	0.076	−0.117	2314
AB BRUX scale sum score	0.485	0.217	0.167	2236	0.027*	0.057	0.912

* Correlation is significant at the 0.05 level (two‐tailed).

## 4 Discussion

OSA is a sleep disorder with a strong impact on public health, predisposing to chronic systemic comorbidities (e.g., hypertension, arrhythmia, stroke, diabetes II type and coronary disease) and being a worsening factor for heart failure and pulmonary hypertension, among others [14]. Unfortunately, despite the potential long‐term consequences of OSA, symptoms (e.g., daytime sleepiness) are often underestimated, and diagnosis is delayed [37]. The prevalence of bruxism among individuals with OSA seems to be higher than in the general population [16, 24], suggesting the existence of a possible correlation between these two sleep‐related conditions [15]. The present study aimed to assess the existence of a correlation between daytime sleepiness and both self‐reported awake and SB in patients with different severities of OSA. Bruxism evaluation was performed by the use of the BRUX scale questionnaire, an effective and simple tool for a general self‐report of bruxism which has been included in the authoritative handbook ‘Principles and Practice of Sleep Medicine’ [35, 38]. Results showed a significant but weak correlation between daytime sleepiness and BRUX scale total sum score (*r* = 0.155; *p* = 0.04), MinSat (*r* = 0.194; *p* < 0.01) and awake BRUX scale sum score (*r* = 0.174; *p* = 0.02). In particular, in the backward multiple linear regression analysis, the awake BRUX scale sum score had a predictive association with daytime sleepiness (*B* = 0.485 [95% CI = 0.076; 0.936]; *p* = 0.027). On the other hand, OSA severity did not show any correlation with any variables.

The relationship between OSA and SB is controversial. Many observational studies showed a higher frequency of SB events in groups of individuals with OSA (35.4%–50.8%) [15, 16, 17, 18, 19, 20, 22] with respect to the general population (12.8 ± 3.1) [3]. In 2018, the consensus paper on bruxism proposed a potential protective effect of SB in individuals with sleep apnoea [2]. This hypothesis was speculated by Manfredini et al. in 2015, who suggested that an increase in masticatory muscle activity, determining a mandibular protrusion and the consequent enlargement of upper airway patency, solved the apnoea event [21]. Moreover, in 2017, Jokubauskas et al. asserted that SB events frequently occurred during micro‐arousal events consequent to apnoea–hypopnoea events and most SB events occurred in temporal conjunction with AH events termination [39].

However, a recent systematic review and meta‐analysis, investigating the chance of occurrence of SB in relationship with different OSA severity, did not confirm this connection and sustained the complexity and the multifactorial aetiology of this relationship [40]. Moreover, current understanding of the aforementioned potential protective effect is suggesting that the relationship is much more complex than believed in the past. Indeed, further studies did not support the one‐to‐one temporal relationship between OSA and SB events [22, 23]. Our findings are in line with such assertions, since no differences in self‐reported bruxism in relation to OSA severity were observed.

However, the present study showed a significant correlation between AB and an OSA‐related symptom as well as a sign of sleep disturbances, viz., daytime sleepiness. This finding supports the hypothesis that the report of bruxism may be a sign of sleep disruption. Evidence has mainly investigated the relation between SB and OSA, and very little information exists on the relation with AB [24]. However, many authors proposed how the relationship between SB and sleep apnoea could be influenced by sleep architecture and the onset of micro‐arousal from sleep. According to Lavigne et al., SB occurs as a rhythmic masseter muscle activity (RMMA) that is the last event of a sequence of physiological events in relation to micro‐arousal [41]. Since sleep apnoea events themselves may cause sleep‐related arousal, bruxism could be indirectly related to sleep apnoea by the occurrence of these autonomic events [24]. Many studies corroborated this statement asserting that the contractions of the masseter muscle after respiratory events were likely dependent on the duration of arousals rather than the occurrence of respiratory events per se [22, 42]. Moreover, Tan et al. claimed that SB individuals showed higher respiratory arousal rates, but a significantly lower spontaneous arousal index [19]. The abovementioned relationship found confirmation in Aarab et al. where mandibular advancement appliance (MAA) therapy significantly reduced jaw‐closing muscle activity time‐related to respiratory arousals in patients with OSA [25].

However, even a direct causal association between SB and micro‐arousal has not been proved yet [43, 44]. In two studies where micro‐arousals were experimentally induced in two groups of healthy individuals with and without bruxism, the authors did not obtain any change in bruxism event frequencies [45, 46]. Therefore, the theory that the relationship between RMMA and micro‐arousal may be modulated by the underlying sleep architecture is worthy of exploration [27].

For these reasons, despite a full understanding being far from achieved, it seems that sleep disruption may have a role in potentially explaining the mutual correlation between sleep‐related conditions. As reported by Macaluso et al., subjects with SB tend to have higher sleep instability with a high dominance of arousal pressure [28]. Moreover, Huang et al. in a questionnaire study found a significant association between self‐reported SB and daytime sleepiness (OR = 2.94 [1.85–4.76]) [47].

The present study enlightens the relevant issue that sleep disturbances may influence the onset of AB. Indeed, self‐reported AB, showing a significant correlation with daytime sleepiness, could be a sign of underlying tiredness and sleepiness in individuals with OSA. The perspective of dental sleep medicine aims to improve the understanding of several sleep‐related conditions for an early recognition of signs and symptoms and prevent possible negative comorbidities. The significant correlation between AB and sleep apnoea may further pave this way and reinforce the role of the dentist as a sentinel for OSA and of bruxism as a medical gateway for dentistry [5].

This study has some limitations. Firstly, sampling was carried out until the beginning of 2020 and was forcedly interrupted by the COVID‐19 pandemic. The weak entity of the correlation between AB and daytime sleepiness, although significant, may depend on the sample size. However, recruitment did not resume after the pandemic to avoid selection bias, since COVID‐19 was shown to have a significant impact on the prevalence of bruxism and psychological distress in the general population [30, 31]. An objective of future studies will be to include larger samples. Secondly, self‐reported assessment for bruxism carries the potential risk of report bias and influences the real prevalence of bruxism [48]. Future studies should include clinical examination and instrumental assessment tools to ensure a comprehensive evaluation of such a multifaceted condition as bruxism. The use of the Standardised Tool for the Assessment of Bruxism (STAB), which is a multidimensional evaluation system that assesses bruxism status, etiological and risk factors, as well as potential consequences [49], and an associated screening version (i.e., BruxScreen) [50] may be useful starting points for getting deeper into the evaluation of comorbidities. The use of the STAB as a point of reference permits physicians to speak the same language, adopting reliable and repetitive tools to assess bruxism and guarantee a constructive comparison between studies. In addition, the use of an ecological momentary assessment (EMA)‐based approach to investigate the association between AB and psychosocial distress should be promoted to collect real‐time data during the day, close in time with the experience in the natural environment [51, 52]. Finally, OSA evaluation was mostly based on the AHI. However, the inclusion of other parameters such as oxygen desaturation index (ODI), apnoea–hypopnoea events duration and respiratory distress index (RDI) could help to increase the knowledge of the nature of the correlation between bruxism and sleep apnoea [53]. Moreover, given the existence of different subtypes of patients with OSA characterised by different primary symptoms and comorbidities [54], in future studies, it is advisable to take into account such variety to delve into the nature of this correlation in detail.

## 5 Conclusions

According to the findings of this study, daytime sleepiness and self‐report AB in individuals with OSA showed a positive but weak correlation. However, OSA severity was not significantly correlated with daytime sleepiness and self‐reported bruxism. AB and sleep disturbances may have a mutual relationship, but future studies with a large sample size and comprehensive assessment of bruxism are recommended.

## Author Contributions

M.P., F.L., R.M.‐R. and D.M. contributed to the conception and design of the study. M.P., L.G.‐N. and R.M.‐R. performed data collection. M.P. and D.M. performed the analysis. M.P., F.L. and D.M. interpreted the results. M.P., L.G.‐N. and D.M. wrote the first draft of the manuscript. All authors critically reviewed the manuscript and approved the final version.

## Ethics Statement

All procedures performed in studies involving human participants were in accordance with the ethical standards of the Advisory Board of the School of Dentistry, University of Padova (Padova, Italy; (#00457‐D87; 26/09/2018)) and with the 1964 Helsinki declaration and its later amendments or comparable ethical standards.

## Consent

Informed consent was obtained from all individual participants included in the study.

## Conflicts of Interest

All authors certify that they have no affiliations with or involvement in any organisation or entity with any financial interest (such as honoraria; educational grants; participation in speakers' bureaus; membership, employment, consultancies, stock ownership, or other equity interest; and expert testimony or patent‐licensing arrangements), or non‐financial interest (such as personal or professional relationships, affiliations, knowledge or beliefs) in the subject matter or materials discussed in this manuscript.

## Data Availability

The data underlying this article will be shared on reasonable request to the corresponding author.
